# Inclusion of Labor Migrants as a Potential Key Population for HIV: A Nationwide Study from Tajikistan

**DOI:** 10.3390/tropicalmed9120304

**Published:** 2024-12-11

**Authors:** Brian Kwan, Hamid R. Torabzadeh, Adebimpe O. Akinwalere, Julie Nguyen, Patricia Cortez, Jamoliddin Abdullozoda, Salomudin J. Yusufi, Kamiar Alaei, Arash Alaei

**Affiliations:** 1 Department of Health Science, College of Health and Human Services, California State University Long Beach, Long Beach, CA 90840, USA; 2 Center for Global Health, College of Health and Human Services, California State University Long Beach, Long Beach, CA 90840, USA; 3 School of Public Health, Brown University, Providence, RI 02912, USA; 4 Tajikistan Ministry of Health and Social Protection of Population, Dushanbe 734000, Tajikistan; 5 National Academy of Science of Tajikistan, Dushanbe 734000, Tajikistan; 6 Institute for International Health and Education, Albany, NY 12207, USA

**Keywords:** HIV, Tajikistan, Central Asia, labor migrants, key populations, regional disparities, rural areas, gender disparities, age, risk factors

## Abstract

Key populations are particularly vulnerable to human immunodeficiency virus (HIV) infection. Nearly half of Tajikistan’s gross domestic product (GDP) originates from labor migrant transfers. While not officially designated as a key population, over 300,000 migrants return to Tajikistan every year at increased risk for HIV due to absence or interruption of treatment, change in risky behaviors, and other factors. We analyzed cross-sectional data from the national registry system operated by the Tajikistan Ministry of Health and Social Protection of individuals (*n* = 10,700) who had been diagnosed with HIV from 1 January 2010 to 30 May 2023. Individual HIV cases resided in five regions: Districts of Republican Subordination (DRS), Dushanbe (Tajikistan’s capital city), Gorno-Badakhshan Autonomous Oblast (GBAO), Khatlon, and Sughd. We developed logistic regression models to investigate the relationships between key population status and demographic characteristics. GBAO has the largest proportion of labor migrants (49.59%), which is much larger than that of the other regions (<32%). In contrast to other key populations, there was a larger proportion of HIV cases in rural areas that were labor migrants (23.25%) in comparison to urban areas (16.05%). In multivariable analysis, the odds of being a labor migrant were 6.248 (95% CI: 4.811, 8.113), 2.691 (95% CI: 2.275, 3.184), and 1.388 (95% CI: 1.155, 1.668) times larger if a case was residing in GBAO, Sughd, or DRS, compared to Dushanbe, respectively. Our research contributes to the field by proposing to expand the definition of key population to include labor migrants in Central Asia who should be emphasized as a vulnerable population at high risk of HIV. We encourage policy action to provide designated HIV funding for labor migrants, increase international attention, and promote potential modifications of national regulations and/or laws regarding prevention and treatment of HIV among non-citizen populations.

## 1. Introduction

The estimated number of people living with HIV (PLHIV) has increased over the last two decades in Tajikistan, from less than 100 people in 2000 to 15,000 in 2022 [1]. The Joint United Nations Programme on HIV/AIDS (UNAIDS) has identified several key populations especially vulnerable to HIV and who frequently lack adequate access to services, including people who inject drugs (PWID), men who have sex with men (MSM), and commercial sex workers (CSW) [2]. While Tajikistan faces challenges and has advanced data-driven interventions among many of these key population groups [3,4,5,6,7,8,9,10], labor migrants may face an increasing burden of HIV in Tajikistan and lack sufficient representation similar to that of other key populations in global HIV infrastructure, research, and funding.

Tajikistan holds the distinction of being the world’s most reliant country on remittances, with 49% of its GDP originating from such transfers [11,12]. Every year, around 40% of the Tajik working-age population emigrates, seeking economic opportunity abroad [13]. According to the Ministry of Labour, Migration and Employment of Tajikistan, over 530,000 citizens left in 2019 to seek employment abroad, primarily in Russia and Kazakhstan [13,14].

While the time range of emigration varies, over 300,000 labor migrants return to Tajikistan every year at increased risk for HIV infection due to the absence or interruption of prevention, change in risky behaviors, and other factors [14,15,16,17]. In Central Asia, mobility has become a key HIV risk factor, with nearly one-fifth of all people newly diagnosed with HIV in Tajikistan in 2018 reporting a recent history of migration and no history of injection drug use [18]. Labor migrants may face a double burden of HIV risk both in the country in which they emigrate and when they return to Tajikistan. Efforts to address this double burden are further complicated by a lack of adequate designated support for labor migrants by Tajikistan’s major HIV service funding streams: The U.S. President’s Emergency Plan for AIDS Relief (PEPFAR) and The Global Fund to Fight AIDS, Tuberculosis and Malaria.

For Tajikistan in particular, Tajik labor migrants with multilevel determinants of HIV risk and testing suffer from inadequate data collection and a limited understanding of the most effective means and interventions for them to access health services [19]. Broadly across all key populations in Tajikistan, the identification of new cases remains difficult, and the capacity of these populations to locate and utilize HIV services warrants further investigation [20]. 

The purpose of this study is to assess the distribution of people living with HIV across various key populations and identify specific associations between different demographic characteristics—such as region, age group, gender, and area (urban/rural status)—and HIV positivity among key populations (including PWID, MSM, CSW, and women or children), and labor migrants as a potential new category. It also explores the inclusion of labor migrants as a potential new key population alongside PWID, MSM, CSW, women, and children to elevate the underrepresented status of labor migrants.

## 2. Materials and Methods

### 2.1. Study Participants 

Our study presents an analysis of cross-sectional data from the national registry system operated by the Tajikistan Ministry of Health and Social Protection, similarly to our prior research [21]. The data include all individuals (*n* = 10,700) who had been diagnosed with HIV by the Tajikistan health system, from 1 January 2010 to 30 May 2023. The Tajikistan Ministry of Health used a combination of enzyme-linked immunosorbent assay (ELISA) and Western blot tests as primary diagnostic tools for HIV prior to 2016. Starting in 2016, the Ministry of Health began utilizing UNAIDS-approved fourth-generation ELISA alongside qualitative polymerase chain reaction (PCR) tests for complex, unclear diagnoses. Individuals resided across five regions: Districts of Republican Subordination (DRS), Dushanbe (Tajikistan’s capital city), Gorno-Badakhshan Autonomous Oblast (GBAO), Khatlon, and Sughd. The study population consists of female and male HIV cases across three age (years) categories (under 19 y, 19–39 y, over 39 y) and in urban and rural areas of residence. Our study explores the inclusion of labor migrants as a potential key population alongside PWID, MSM, CSW, and women or children.

### 2.2. Statistical Analysis

We investigated the frequency (%) of HIV cases of key population status (i.e., PWID, MSM, CSW, women or children, labor migrants) in each category of a demographic characteristic (i.e., Region of Residence, Age Group, Gender, Area of Residence). We conducted chi-squared and Fisher’s exact tests to test for associations between each key population status and each demographic characteristic. Logistic regression models were developed with the binary outcome of whether a HIV case is of a key population status (i.e., Yes vs. No), with demographic characteristics as predictors. Both simple (single predictor) and multivariable (multiple predictor) models were developed to examine the direction and magnitude of associations between predictor and outcome. Odds ratio estimates as effect sizes and their 95% confidence intervals were provided for quantifying these associations. *P*-values were examined for statistical significance at the 5% level. A notable advantage in comparing both the simple and multivariable models is examining for changes in effect size and statistical significance after adjusting for other influential factors for the outcome. All statistical analysis was conducted using the R programming language, version 4.4.0, Vienna, Austria [22].

## 3. Results

We provided descriptive statistics on the demographic characteristics (i.e., region of residence, age group, gender, area of residence) of the HIV cases in Tajikistan from 2010 to 2023 in our prior work (*n* = 10,700) [21]. Approximately 96% of the HIV cases were from the regions of DRS, Dushanbe, Khatlon, and Sughd in Tajikistan, with each region contributing at least 20% of cases. Moreover, we identified HIV cases who are members of the key populations: PWID, MSM, CSW, women or children, and labor migrants. There were 1801 (16.83%) who were PWID, 192 (1.79%) who were MSM, 489 (4.57%) who were CSW, 5281 (49.36%) who were either a woman or a child, and 2146 (20.06%) who were labor migrants.

In Table 1, we present the frequency (%) of HIV cases of key population status (i.e., PWID, MSM, CSW, women or children, labor migrants) in each category of Region of Residence, Age Group, Gender, and Area of Residence. 

The rightmost column of Table 1 examined HIV cases identified as labor migrants. There was a notable disparity in the proportion of HIV cases who were labor migrants in each region. GBAO had the largest proportion of labor migrants (49.59%), which was much larger than that of the region with the next highest proportion of labor migrants, Sughd, with 31.37% of HIV cases as labor migrants. Dushanbe and Khatlon consisted of the lowest proportion of labor migrants, falling below 15%. The age groups 19–39 y and >39 y had over 20% of its HIV cases who were labor migrants. Moreover, greater than a quarter of male HIV cases were labor migrants (28.83%), which is greater than three times the proportion of female HIV cases who were labor migrants (7.91%). Finally, in contrast to other key populations, a larger proportion of HIV cases in rural areas were labor migrants (23.25%) in comparison to urban areas (16.05%).

The second to the rightmost column of Table 1 examined HIV cases who were women or children. Close to or approximately 50% of HIV cases were women or children in nearly all of the categories for region of residence and area of residence, with the exception of GBAO, with women or children accounting for 41.05% of its HIV cases. Women or children accounted for 45.1% of HIV cases in the 19–39 y age group and 35.63% in the >39 y age group.

The middle column of Table 1 examined HIV cases who were CSW. In looking at each category for region of residence, we see that there is an approximate range from 2% to 6% of HIV cases in each region who identified as CSW, with Khatlon having the greatest and Sughd having the lowest proportions of CSW HIV cases. Among HIV cases for the age groups of 19–39 y and >39 y, approximately 4% to 6% were CSW. The proportion of female HIV cases who were CSW (9.68%) was greater than 10 times that of male HIV cases who were CSW (0.84%). Among HIV cases in both urban and rural residences, 5.42% and 3.92% were CSW, respectively.

The second to the leftmost column of Table 1 examined HIV cases who were MSM. The proportion of HIV cases who identified as MSM were less than 4% in every specific category of region of residence, age group, gender, and area of residence. When looking at just male HIV cases, there were 3.1% who were MSM.

The leftmost column of Table 1 examined HIV cases who were PWID. Among HIV cases residing in each of the regions in Tajikistan, greater than a quarter of HIV cases identified as PWID in Dushanbe and GBAO, at 28.22% and 31.4%, respectively. A larger proportion of HIV cases in the >39 y age group (23.5%) were PWID compared to those in the 19–39 y (17.36%) and <19 y (0.38%) age groups. The proportion of male HIV cases who were PWID (27.55%) was greater than 10 times that of female HIV cases who were PWID (2.15%). Over a quarter of HIV cases residing in urban areas were PWID (26.46%), which was greater than twice the proportion of those living in rural areas (9.38%).

Furthermore, we conducted chi-squared and Fisher’s exact tests to test for associations between each key population status and each demographic characteristic. All key population statuses, including labor migrants, were significantly associated with each demographic characteristic at the 5% level (most were *p* < 0.001), with the exception of the key population status of women or children with area of residence (*p* = 0.901).

In Table 2, we provide several fitted simple logistic regression models with each demographic variable as a predictor for the binary outcome of whether an HIV case belongs to a key population (i.e., PWID, MSM, CSW, women or children, labor migrants).

Region of residence as a predictor for each of the five key population status outcomes is shown in part (a) of Table 2, with Dushanbe serving as the reference group. Additionally, the model with labor migrant status as the outcome has the largest effect sizes in the odds ratio with region of residence as a predictor. The odds of an HIV case being a labor migrant were 6.605 (95% CI: 5.22, 8.356) times larger if a case was residing in GBAO compared to Dushanbe. In addition, the odds were 3.078 (95% CI: 2.669, 3.551) and 1.757 (95% CI: 1.507, 2.05) times larger if a case was residing in Sughd and DRS, compared to Dushanbe, respectively. These odds ratio estimates were statistically significant at the 5% level (*p* < 0.001) and were larger than nearly all other odds ratio estimates in the models for PWID, MSM, CSW, and women or children in part (a). In looking at parts (b, d) in Table 2, we see that the odds of an HIV case being a labor migrant were 1.396 (95% CI: 1.258, 1.549) times larger if an HIV case was >39 y compared to being <39 y and 1.587 (95% CI: 1.438, 1.751) times larger if an HIV case was residing in a rural area compared to an urban area.

In Table 3, we provide five fitted multivariable logistic regression models, with each utilizing all our aforementioned demographic variables as predictors for each of the five key population status outcomes. Each model uses region of residence as a primary predictor and adjusts for age group, gender, and area of residence.

In the rightmost column of Table 3, we see that the odds of an HIV case being a labor migrant were 6.248 (95% CI: 4.811, 8.113) times larger if an HIV case was residing in GBAO compared to Dushanbe. In addition, the odds were 2.691 (95% CI: 2.275, 3.184) and 1.388 (95% CI: 1.155, 1.668) times larger if an HIV case was residing in Sughd and DRS, compared to Dushanbe, respectively. These odds ratio estimates were statistically significant at the 5% level (*p* < 0.001). The odds ratio estimates for GBAO and Sughd relative to that of Dushanbe were larger than nearly all other odds ratio estimates in the models for PWID, MSM, CSW, and women or children. Additionally, the direction and magnitude of associations for the multiple predictors of the multivariable labor migrant model were largely consistent with using the same predictors for the simple labor migrant models from Table 2 (parts a–d).

## 4. Discussion

### 4.1. Labor Migrants Require Renewed Focus as a New Key Population

Other than women and children, labor migrants were the largest single key population identified among HIV cases in Tajikistan from 2010 to 2023. In addition to broad disparities in likelihood of being any given HIV-positive key population in Tajikistan, our findings reveal unique disparities in the demographic characteristics that increase or decrease the likelihood of being an HIV-positive labor migrant in Tajikistan. 

In multivariable models (adjusted for multiple predictors), compared to the capital city Dushanbe, HIV-positive labor migrants were more likely to be from DRS, Sughd, and GBAO and from rural areas, findings which are mostly the inverse of those for other key populations. Compared to other key populations, with the exception of PWID (in GBAO), HIV-positive labor migrants were more likely to reside in regions other than the capital city, Dushanbe. For instance, all HIV-positive key populations were less likely to reside in Sughd, with the exception of HIV-positive labor migrants, who had nearly three times the odds of being from Sughd compared to Dushanbe. This finding requires further research, as Sughd generally excels in HIV prevention among certain key populations compared to other regions, which may be a result of its substantial local economy as a percentage of all new job growth in Tajikistan [23]. On the other hand, GBAO has been well-documented to be particularly more vulnerable to HIV-positive labor migrants due to longer migration timeliness and less frequent visits home [15].

While the overall risk of HIV among key populations is greater in urban areas, as evidenced by our findings, our findings reveal that labor migrants in Tajikistan are a key exception who are more likely to be from rural areas of the country. Labor migrants and their families are more likely to reside in hard-to-reach rural areas that do not receive proportional levels of international HIV funding [23]. Further, many international donors, including Global Fund/PEPFAR, do not prioritize funding to labor migrants or their families like they do for more broadly recognized key populations like PWID, MSM, and CSW. 

Labor migrants face unique HIV barriers that warrant their designation as a key population. For every year spent in Russia, late presentation for a Tajik labor migrant with HIV increases by 4.0% [24]. Limited awareness regarding HIV risk was identified among Tajik labor migrants upon their return home [25]. As a result, they may engage in behaviors that put them at higher risk of acquiring HIV or spreading it, such as unprotected sexual activity or sharing needles for drug use. Further, there has been an increase in HIV cases noted among the spouses of male Tajik labor migrants after their return [25]. This suggests that HIV may be transmitted from labor migrant husbands to their wives upon their return home. 

A study conducted in the UK on African migrants has shown that the main barriers to HIV testing among migrants are rooted in a lack of knowledge and understanding, worsened by being newcomers in a foreign healthcare system and facing different cultural norms [26]. This gap in knowledge significantly affects migrants’ willingness and ability to access HIV testing services [27]. Further, diaspora labor migrants avoid undergoing HIV testing due to fear of being deported, jeopardizing their current employment, or facing stigma and social exclusion if they are found to be HIV-positive [28]. Similarly, migrants in the Netherlands, particularly those from African and Afro-Caribbean communities, experience significant anxiety about the potential repercussions of an HIV diagnosis. They are particularly concerned about the social stigma and the potential public exposure of their status within their close-knit communities [29].

Labor migrants who are unaware of their HIV-positive status do not receive necessary treatment until they experience worsening symptoms that prompt them to seek medical care, at which time the HIV infection may have already significantly compromised their health, and/or when they return to Tajikistan [15]. Targeted HIV education and prevention efforts aimed at migrant populations, particularly before and after their return, can help reduce the risk of HIV transmission both abroad and upon their return to Tajikistan. 

Overall, our findings point to a diverse set of demographic variables associated with HIV positivity among labor migrants (namely, regions and rurality) that largely contrast with the associations among other key populations. These findings support why a designation of key population for labor migrants and their families may be essential to addressing their unique demographic associations with HIV infection.

### 4.2. People Who Inject Drugs (PWID) Require Localized HIV Prevention and Care

With a population size estimated at 23,100, PWID and their sexual partners continue to be a leading demographic group impacted by HIV in Tajikistan [30]. By the end of 2018, data from the national HIV program indicated that around 30% of individuals in Tajikistan had either a past history of injecting drugs or were presently engaged in injecting drugs [30]. Further, official statistics may not fully capture the extent of injection drug use, as in other neighboring countries [31]. 

In multivariable models (adjusted for multiple predictors), compared to the capital city Dushanbe, HIV-positive PWID were more likely to be from GBAO and less likely to be from Khatlon, Sughd, and rural areas. These findings are supported by previous research that indicates potential cross-over effects between PWID and labor migrants in GBAO. A prior study demonstrated that labor migrants from Dushanbe had the lowest rates of syringe sharing, while those from the GBAO had the highest rates [15]. Further, the PWID group is the only key population (other than women or children) with a significantly varied association with infection based upon age. Those greater than 39 years of age have nearly two times the odds of being an HIV-positive PWID compared to those less than or 39 years of age. This finding further supports our prior finding that the older population may face distinct challenges in preventing HIV transmission in Tajikistan [21]. 

Like many peer countries, PWID face challenges in preventing HIV infection and accessing timely care [32]. Stigma and discrimination associated with illegal drug use present substantial barriers for individuals seeking HIV prevention, care, and treatment services in many countries, and as a result, lead to delays in diagnosis, inadequate treatment, and poor health outcomes [33,34]. Within Central Asian nations, stigma and discrimination persist among both health service providers and the general population. This, coupled with mandatory registration of PWID and a history of criminalization of drug use, contribute to high rates of reluctance to engage with healthcare facilities, potentially exacerbating the spread of HIV within this population and hindering efforts to control the epidemic [35,36,37,38].

Despite these well-documented challenges, our findings demonstrate how major international funding and HIV prevention programs, specifically targeted to regions including Dushanbe, Khatlon, and Sughd, can reduce the risk of HIV infection compared to their respective counterparts [39]. Future efforts must be focused on those from GBAO and those greater than 39 years of age.

### 4.3. Commercial Sex Workers (CSW) Have Low Overall HIV Prevalence and Can Benefit from Targeted Interventions

The commercial exchange of sex has a role in the HIV epidemic. The prevalence of HIV among CSWs is disproportionately high, underscoring the urgent need to rapidly expand interventions aimed at preventing new HIV infections [40,41]. Generally, structural risk factors indirectly increase the vulnerability of sex workers to HIV infection by limiting their access to preventive healthcare and HIV services [42,43,44]. Further, a substantial overlap between sex work and injection drug use activities has been observed, with high injection drug use prevalence among CSW communities [45,46]. These combined risk factors may play a role in the early detection of CSW over the time period.

The prior literature indicates that HIV prevalence among Tajikistani female sex workers (FSW) is comparatively low, but also highlights how stigma against FSWs was identified as a barrier to HIV testing, with over 60% of FSW reporting such instances [6]. Research studies indicate that HIV testing conducted within communities results in greater testing uptake compared to testing within healthcare facilities. The primary obstacles identified for HIV testing include perceived low risk, apprehension and concerns, limited access to healthcare services, healthcare provider hesitation to administer the test, and a lack of financial and personnel resources [47]. 

Overall, our findings indicate a generally low prevalence of HIV infection among CSW (particularly compared to other key populations in Tajikistan). In adjusted models among CSW, in Sughd, the odds of being HIV-positive were roughly one-third that of Dushanbe, demonstrating clear areas where resources must be diverted in preventing HIV infection within this group.

### 4.4. Men Who Have Sex with Men (MSM) Require Integration Within Health Care System

Studies estimate HIV prevalence among MSM ranges from 1% to 2% in Central Asian countries, including Kazakhstan, Kyrgyzstan, and Tajikistan [48,49]. Our adjusted findings indicate no specific associations between demographic characteristics and MSM HIV positivity in Tajikistan, other than some regional differences. In adjusted models, among MSM, those who reside in DRS and Sughd have around half the odds of being HIV-positive compared to those from Dushanbe.

The social stigma surrounding homosexuality in Tajikistan may create barriers to accessing healthcare and social services for this population [50]. In Tajikistan, unfavorable attitudes towards HIV testing, such as the perception that testing is only for those engaging in promiscuous behavior, can contribute to the low utilization of testing services [51]. Services solely targeted at MSM may not be attractive to men who are concerned about being publicly recognized as part of this group [52]. Since homosexuality was legalized in the late 1990s in Tajikistan, there has been some progress in reducing the social stigma of male homosexuality. Integrating services for MSM within services for the normal population, along with providing sensitivity training to healthcare professionals, could help facilitate culturally sensitive care in a non-discriminatory setting, encouraging testing, treatment initiation, and continuous engagement in HIV care [53].

While our findings do not indicate any significant associations among specific demographic groups, substantial prior research has shown how Tajikistan labor migrants, including those who are MSM, may be more at risk for risky sexual behavior while abroad [10,54]. A similar focus on hotspots of HIV positivity among labor migrants may help reduce the burden of HIV among MSM in this nation.

### 4.5. Limitations

There are some limitations to consider in this study. First, we utilize cross-sectional data from the Tajikistan Ministry of Health and Social Protection that only include diagnosed clinical cases reported by the Tajikistan health system. However, it is important to note that our findings largely parallel and validate epidemiological data in the prior literature. Secondly, observations may overlap among more than one key population category. Thirdly, key population designations are largely self-reported, and some observations may not disclose their risky behaviors. Finally, we included 2020 HIV data that were adversely affected by the start of the COVID-19 pandemic.

### 4.6. Next Steps

In December 2023, the Plenum of the Supreme Court of Tajikistan marked a significant step towards decriminalization of HIV exposure and transmission, allowing Tajik courts to more objectively examine issues related to criminal liability for HIV exposure and transmission under Article 125 of the Criminal Code [55]. This may have trickle-down effects in both preventing HIV infection and disease progression through robust testing and protecting the human rights of PLHIV, and underscores Tajikistan’s commitment to ending the HIV epidemic.

While this is a positive step, our findings separately point to how different populations experience HIV infection and why targeted efforts among different groups, coupled with a renewed international focus on addressing the unique needs and challenges of labor migrants, can make a key impact in Tajikistan.

## 5. Conclusions

Our research contributes to the field by proposing to expand the definition of key populations to include labor migrants in Central Asia who should be emphasized as a vulnerable population at high-risk of HIV and designated for allocated HIV funding and international support. Labor migrants face unique challenges that contrast with those of the other major key populations in Tajikistan, including increased HIV infection across multiple diverse regions and rural communities. The international community should proactively address the role of this large and growing key population in driving HIV infections through a combination of financing, policy, and research. Specific policy action is encouraged at both the national (i.e., potential modifications of national regulations and/or laws regarding prevention and treatment of HIV among non-citizen populations) and international level (i.e., designated allocation of HIV prevention and treatment funding for labor migrants and further data collection across national borders). Specific research action is encouraged to better understand the journey of labor migrants and their unique interactions with the workforce, healthcare system, and society in both their home nation and nation of labor migration. Our findings point to the importance of targeted HIV interventions unique to each key population, including labor migrants where the HIV burden seems to be higher. Further, we hope that our analysis better informs other nations’ targeted research and interventions to reach key populations.

## Figures and Tables

**Table 1:** Frequency (%) of HIV cases of key population status (i.e., People Who Inject Drugs (PWID), Men Who Have Sex With Men (MSM), Commercial Sex Workers (CSW), Women or Children, Labor Migrants) in each category of a demographic characteristic (i.e., Region of Residence, Age Group, Gender, Area of Residence). Example interpretation: There were 340 (or 12.88% of) cases residing in the region of Dushanbe who were labor migrants. *p*-values (*p*) for the associations between a key population status and demographic characteristic (e.g., *p* < 0.001 for Labor Migrant and Region of Residence) were obtained from chi-squared and Fisher’s exact tests.

Variable	PWID	MSM	CSW	Women or Children	Labor Migrants
**Region of Residence**	*p* < 0.001	*p* < 0.001	*p* < 0.001	*p* < 0.001	*p* < 0.001
Districts of Republican Subordination (DRS)	328 (14.86)	29 (1.31)	91 (4.12)	1094 (49.57)	453 (20.53)
Dushanbe	745 (28.22)	71 (2.69)	155 (5.87)	1348 (51.06)	340 (12.88)
Gorno-Badakhshan Autonomous Oblast (GBAO)	114 (31.4)	0 (0)	11 (3.03)	149 (41.05)	180 (49.59)
Khatlon	329 (10.8)	64 (2.1)	184 (6.04)	1574 (51.66)	415 (13.62)
Sughd	277 (11.49)	28 (1.16)	46 (1.91)	1100 (45.64)	756 (31.37)
**Age Group**	*p* < 0.001	*p* < 0.001	*p* < 0.001	*p* < 0.001	*p* < 0.001
<19 y	5 (0.38)	3 (0.23)	7 (0.53)	1313 (99.02)	2 (0.15)
19–39 y	1151 (17.36)	140 (2.11)	367 (5.54)	2990 (45.1)	1478 (22.3)
>39 y	645 (23.5)	49 (1.79)	115 (4.19)	978 (35.63)	666 (24.26)
**Gender**	*p* < 0.001	NA	*p* < 0.001	NA	*p* < 0.001
Female	97 (2.15)	0 (0)	437 (9.68)	4516 (100)	357 (7.91)
Male	1704 (27.55)	192 (3.1)	52 (0.84)	765 (12.37)	1789 (28.93)
**Area of Residence**	*p* < 0.001	*p* = 0.007	*p* < 0.001	*p* = 0.901	*p* < 0.001
Rural	563 (9.38)	89 (1.48)	235 (3.92)	2957 (49.29)	1395 (23.25)
Urban	1235 (26.46)	103 (2.21)	253 (5.42)	2307 (49.43)	749 (16.05)

**Table 2:** Simple logistic regression models (one predictor only) with binary outcome for a key. population status (i.e., People Who Inject Drugs (PWID), Men Who Have Sex With Men (MSM), Commercial Sex Workers (CSW), Women or Children, Labor Migrants). Each key population outcome model contains a single predictor that is a demographic characteristic (i.e., Region of Residence, Age Group, Gender, Area of Residence). Part (a)—Region of Residence as a predictor, Part (b)—Age Group as a predictor, Part (c)—Gender as a predictor, Part (d)—Area of Residence as a predictor. OR = Odds Ratio, CI = Confidence Interval, Ref = Reference Group.

(a). Region of Residence										
	PWID		MSM		CSW		Women or Children		Labor Migrants	
	OR (95% CI)	*p*-Value	OR (95% CI)	*p*-Value	OR (95% CI)	*p*-Value	OR (95% CI)	*p*-Value	OR (95% CI)	*p*-Value
**Region of Residence**										
Dushanbe (Ref.)	—	—	—	—	—	—	—	—	—	—
Districts of Republican Subordination (DRS)	0.444 (0.384, 0.513)	<0.001	0.482 (0.312, 0.745)	0.001	0.689 (0.529, 0.899)	0.006	0.942 (0.841, 1.055)	0.301	1.757 (1.507, 2.05)	<0.001
Gorno-Badakhshan Autonomous Oblast (GBAO)	1.165 (0.919, 1.476)	0.208	NA	NA	0.501 (0.269, 0.933)	0.029	0.667 (0.534, 0.834)	<0.001	6.605 (5.22, 8.356)	<0.001
Khatlon	0.308 (0.267, 0.355)	<0.001	0.776 (0.551, 1.093)	0.147	1.03 (0.827, 1.284)	0.79	1.024 (0.923, 1.137)	0.653	1.063 (0.911, 1.24)	0.435
Sughd	0.33 (0.284, 0.384)	<0.001	0.425 (0.274, 0.661)	<0.001	0.312 (0.223, 0.436)	<0.001	0.805 (0.72, 0.899)	<0.001	3.078 (2.669, 3.551)	<0.001
**(b). Age Group**										
**Age Group**										
<39 y (Ref.)	—	—	—	—	—	—	—	—	—	—
>39 y	1.806 (1.621, 2.013)	<0.001	0.993 (0.716, 1.377)	0.966	0.886 (0.716, 1.097)	0.268	0.47 (0.429, 0.514)	<0.001	1.396 (1.258, 1.549)	<0.001
**(c). Gender**										
**Gender**										
Male (Ref.)	—	—	—	—	—	—	—	—	—	—
Female	0.058 (0.047, 0.071)	<0.001	NA	NA	12.634 (9.451, 16.888)	<0.001	NA	NA	0.21 (0.186, 0.238)	<0.001
**(d). Area of Residence**										
**Area of Residence**										
Urban (Ref.)	—	—	—	—	—	—	—	—	—	—
Rural	0.288 (0.258, 0.321)	<0.001	0.667 (0.501, 0.888)	0.006	0.711 (0.593, 0.853)	<0.001	0.994 (0.921, 1.073)	0.885	1.587 (1.438, 1.751)	<0.001

**Table 3:** Multivariable logistic regression models (multiple predictors) with binary outcome for a key population status (i.e., People Who Inject Drugs (PWID), Men Who Have Sex With Men (MSM), Commercial Sex Workers (CSW), Women or Children, Labor Migrants). Each key population outcome model contains all demographic characteristics (i.e., Region of Residence, Age Group, Gender, Area of Residence) as predictors. aOR = Adjusted Odds Ratio, CI = Confidence Interval, Ref = Reference Group.

	PWID		MSM		CSW		Women or Children		Labor Migrants	
	aOR (95% CI)	*p*-Value	aOR (95% CI)	*p*-Value	aOR (95% CI)	*p*-Value	aOR (95% CI)	*p*-Value	aOR (95% CI)	*p*-Value
**Region of Residence**										
Dushanbe (Ref.)	—	—	—	—	—	—	—	—	—	—
Districts of Republican Subordination (DRS)	0.891 (0.742, 1.069)	0.215	0.551 (0.333, 0.915)	0.021	0.835 (0.608, 1.146)	0.265	0.932 (0.815, 1.066)	0.304	1.388 (1.155, 1.668)	<0.001
Gorno-Badakhshan Autonomous Oblast (GBAO)	2.03 (1.523, 2.705)	<0.001	NA	NA	0.572 (0.3, 1.09)	0.089	0.753 (0.597, 0.95)	0.017	6.248 (4.811, 8.113)	<0.001
Khatlon	0.621 (0.521, 0.741)	<0.001	0.886 (0.579, 1.355)	0.577	1.167 (0.885, 1.539)	0.274	1.011 (0.892, 1.146)	0.866	0.856 (0.713, 1.028)	0.097
Sughd	0.522 (0.437, 0.624)	<0.001	0.471 (0.288, 0.768)	0.003	0.339 (0.236, 0.487)	<0.001	0.848 (0.748, 0.963)	0.011	2.691 (2.275, 3.184)	<0.001
**Age Group**										
<39 y (Ref.)	—	—	—	—	—	—	—	—	—	—
>39 y	1.762 (1.557, 1.994)	<0.001	1.076 (0.774, 1.495)	0.663	1.15 (0.919, 1.438)	0.221	0.476 (0.434, 0.521)	<0.001	1.105 (0.987, 1.237)	0.083
**Gender**										
Male (Ref.)	—	—	—	—	—	—	—	—	—	—
Female	0.052 (0.042, 0.065)	<0.001	NA	NA	12.811 (9.573, 17.145)	<0.001	NA	NA	0.195 (0.172, 0.222)	<0.001
**Area of Residence**										
Urban (Ref.)	—	—	—	—	—	—	—	—	—	—
Rural	0.286 (0.248, 0.329)	<0.001	0.828 (0.571, 1.202)	0.321	0.724 (0.572, 0.917)	0.007	1.019 (0.925, 1.122)	0.706	1.486 (1.309, 1.686)	<0.001

## Data Availability

The data that support the findings of this study are the property of the Ministry of Health of Tajikistan and are not publicly available.
